# Spectral CT Hybrid Images in the Diagnostic Evaluation of Hypervascular Abdominal Tumors—Potential Advantages in Clinical Routine

**DOI:** 10.3390/diagnostics11091539

**Published:** 2021-08-25

**Authors:** Timo Alexander Auer, Felix Wilhelm Feldhaus, Laura Büttner, Martin Jonczyk, Uli Fehrenbach, Dominik Geisel, Georg Böning

**Affiliations:** 1 Department of Radiology, Campus Virchow-Klinikum, Charité—Universitätsmedizin Berlin, Corporate Member of Freie Universität Berlin, Humboldt-Universität zu Berlin, and Berlin Institute of Health, 10178 Berlin, Germany; timo-alexander.auer@charite.de (T.A.A.); felix.feldhaus@charite.de (F.W.F.); laura.buettner@charite.de (L.B.); martin.jonczyk@charite.de (M.J.); uli.fehrenbach@charite.de (U.F.); dominik.geisel@charite.de (D.G.); 2 Berlin Institute of Health (BIH), Anna-Louisa-Karsch 2, 10178 Berlin, Germany

**Keywords:** hybrid imaging, spectral CT (SCT), gemstone spectral imaging (GSI), neuroendocrine neoplasms (NENs), hepatocellular carcinoma (HCC), renal cell carcinoma (RCC), efficiency

## Abstract

Background: This study aimed to investigate the use of spectral computed tomography (SCT) hybrid images combining virtual monoenergetic images (VMIs) and iodine maps (IMs) as a potentially efficient search series for routine clinical imaging in patients with hypervascular abdominal tumors. Methods: A total of 69 patients with hypervascular abdominal tumors including neuroendocrine neoplasms (NENs, *n* = 48), renal cell carcinoma (RCC, *n* = 10), and primary hepatocellular carcinoma (HCC, *n* = 11) were analyzed retrospectively. Two radiological readers (blinded to clinical data) read three CT image sets (1st a reference set with 70 keV; 2nd a 50:50 hybrid 140 keV/40 keV set; 3rd a 50:50 hybrid 140 keV/IM set). They assessed images subjectively by rating several parameters including image contrast, visibility of suspicious lesions, and diagnostic confidence on five-point Likert scales. In addition, reading time was estimated. Results: Median subjective Likert scores were highest for the 1st set, except for image contrast, for which the 2nd set was rated highest. Scores for diagnostic confidence, artifacts, noise, and visibility of suspicious lesions or small structures were significantly higher for the 1st set than for the 2nd or 3rd set (*p* < 0.001). Regarding image contrast, the 2nd set was rated significantly higher than the 3rd set (*p* < 0.001), while the median did not differ significantly compared with the 1st set. Agreement between the two readers was high for all sets. Estimated potential reading time was the same for hybrid and reference sets. Conclusions: Hybrid images have the potential to efficiently exploit the additional information provided by SCT in patients with hypervascular abdominal tumors. However, the use of rigid weighting did not significantly improve diagnostic performance in this study.

## 1. Introduction

Neuroendocrine neoplasms (NENs), renal cell carcinoma (RCC), and primary hepatocellular carcinoma (HCC) are typically hypervascular in arterial-phase contrast-enhanced computed tomography (CT) [1,2,3]. Detection and correct characterization of these hypervascular abdominal lesions is crucial for patient prognosis and therapy monitoring.

Spectral CT (SCT) contains more information than equivalent single-energy scans. However, using this information efficiently and finding the best balance in terms of image quality, reading time, and ease of interpretation is challenging. The use of spectral SCT-based virtual monoenergetic images (VMIs) and iodine maps (IMs) significantly improves the detection of tumor lesions [4,5,6,7,8,9,10]. Furthermore, VMIs and IMs have shown some potential to improve the diagnostic performance of CT in the differentiation of several types of tumors and pathologies and therapy monitoring in these patients [11,12,13,14]. However, it is not possible to efficiently assess all kinds of images, as reading is too complex and time-consuming for clinical routine [15]. A solution to overcome this limitation could be to characterize lesions based on their Hounsfield unit (HU) slope to be extracted from VMIs [13,16]. While low keV levels are favored for detection of hypervascular lesions in the literature [15,17,18,19], one must be aware that low keV levels increase image noise, which in turn affects sensitivity, especially for detection of small lesions. A solution could be a hybrid approach combining the high contrast of low-keV images and the acceptable noise levels of high-keV images. Alternatively, one could combine the acceptable noise level of high-keV images and the iodine concentration directly available from IMs.

The aim of this study was to evaluate and compare different hybrid image datasets in terms of subjective diagnostic confidence and image quality in the diagnosis of hypervascular abdominal tumors (NEN, RCC, and HCC).

## 2. Material and Methods

### 2.1. Patient Population and Study Design

**Figure 1:**
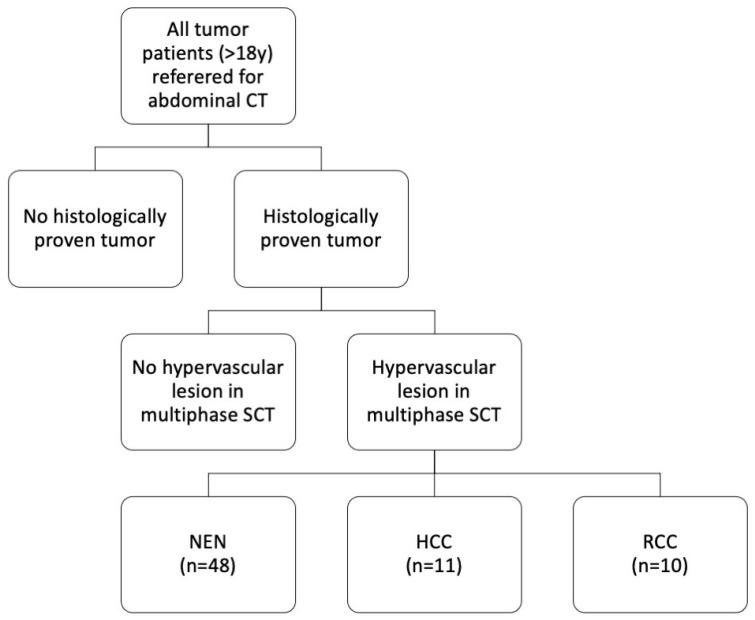
Study cohort. Sixty-nine patients with histologically proven tumors and hypervascular liver lesions in a multiphase abdominal SCT scan were included. SCT: spectral computed tomography, NEN: neuroendocrine neoplasm, HCC: hepatocellular carcinoma, RCC: renal cell carcinoma.

For this retrospective single-center study, written informed consent was waived, and all patient data were recorded and stored anonymously. The patients included in our study underwent SCT examinations at our department from March 2016 to July 2017. Inclusion criteria were: age > 18 years and histologically proven tumor with at least one visible hypervascular lesion in the multiphase abdominal SCT scan (Figure 1). Patients with contraindications to CT or contrast agent (CA) administration were excluded.

### 2.2. CT Scanner and Protocol

The examinations were performed, as described before, on a second-generation spectral multislice CT scanner (Revolution HD, GE Healthcare, Milwaukee, WI, USA) [20]. After creation of a posterior-anterior scout and intravenous CA injection, multiphase scans with identical scan ranges for late arterial and portal venous phases were acquired, followed by a venous phase. The arterial phase was acquired in SCT mode using GE’s ultra-fast kV switching between 80 and 140 kV, known as Gemstone Spectral Imaging (GSI). The portal venous and venous phases were acquired in standard single-energy mode.

For contrast-enhanced imaging, a nonionic contrast agent (Xenetix 350^®^, Guerbet, Villepinte, France) was injected at a dose of 1.5 mL/kg body weight (up to a total dose of 120 mL) with a flow rate of 4 mL/s, followed by an isotonic saline flush at a flow rate of 4 mL/s. A bolus tracking software (threshold of 150 HU in the abdominal descending aorta; SmartPrep, GE Healthcare, Milwaukee, WI, USA) was used. Our standard clinical protocol with delay times set to 18 s (late arterial), 53 s (portal venous), and 133 s (venous) was used.

Postprocessing was performed with the soft tissue kernel to reconstruct images from raw data. Using GE’s GSI Volume Viewer, we generated VMIs (40 keV and 140 keV), iodine maps, and hybrid overlays with 50% blending. Images were reconstructed with 0.625 mm slice thickness. Further technical parameters are compiled in Table 1.

Identical CT scan parameters were used for acquisition of arterial, portal venous, and venous phases except for the spectral mode, tube current limits, and auto mA. The tool routinely utilized in the user interface to set the desired image quality in GE CT systems is called noise index (NI). The NI is compared with the attenuation measured in the preliminary CT scout and referenced to the standard deviation in radiodensity (HU) in a specific-sized water phantom. Auto mA is *z*-axis-based automated tube current modulation, which is not available from the vendor for the spectral mode. In spectral mode, limitations of average mA can be preset, while, in the single-energy mode, min/max mA can be preset. DFOV is display field of view, and SFOV is scan field of view.

### 2.3. Subjective Scoring and Measurements

Two radiologists with twelve and seven years of experience independently read three different image sets:

First set: A reference set with 70 keV;

Second set: A hybrid 140 keV/40 keV set with 50:50 blending;

Third set: A hybrid 140 keV/iodine map set with 50:50 blending.

The two readers were blinded to clinical data and evaluated the following subjective parameters on five-point Likert scales, as described before [21]:

Level of diagnostic confidence (5 = full confidence; 4 = high confidence; 3 = confidence only for a limited clinical entity; 2 = poor confidence; 1 = no confidence).

Artifacts (5 = no artifacts; 4 = minor artifacts not interfering with diagnostic decision making; 3 = minor artifacts affecting visualization of minor structures but diagnosis still possible; 2 = major artifacts affecting visualization of major structures but diagnosis still possible; 1 = artifacts affecting diagnostic information).

Image noise (5 = minimal image noise; 4 = less than average noise; 3 = average noise; 2 = above-average noise; 1 = unacceptable image noise).

Image contrast (5 = excellent visualization; 4 = above-average contrast; 3 = acceptable image contrast; 2 = suboptimal image contrast; 1 = poor image contrast).

Visibility of suspicious lesions (5 = excellent visualization; 4 = above-average visibility; 3 = acceptable image visibility; 2 = suboptimal image visibility; 1 = unacceptable visualization).

Visibility of small anatomical structures (5 = excellent visualization; 4 = above-average visibility; 3 = acceptable image visibility; 2 = suboptimal image visibility; 1 = unacceptable visualization).

Likert scores are presented as medians and their interquartile ranges (IQR) [22].

Furthermore, in each patient, maximum abdominal diameter in sagittal and coronal orientation and tumor area of largest visible liver lesion were measured in the reference set by a third radiologist with two years of experience.

### 2.4. Estimated Reading Time

The estimated reading time for image interpretation was calculated as described elsewhere [15]. Briefly, the reading time for the hybrid series was calculated from the number of images that had to be taken into consideration. For comparison, reading time was estimated in the same way for all possible DECT image series. Assuming the same reading intensity for all datasets, one can directly correlate the number of images and reading time. As the standard reading process includes at least thin (0.625 mm) axial images, the total number of thin slices to be read was defined as the standard of reference for estimating reading time.

### 2.5. Statistics

Statistical analysis was performed using SPSS Software v. 24 (IBM; Armonk, NY, USA). All values, unless otherwise indicated, are provided as median/IQR. Graphics were created with GraphPad Prism v.5 (GraphPad Software, San Diego, CA, USA). Variance analysis was performed using Friedmann 2-facor variance analysis by ranks and corrected Dunn–Bonferroni post hoc tests. For inter-reader correlation, Kendall’s tau-b was calculated. A *p* < 0.05 was considered statistically significant.

## 3. Results

### 3.1. Study Population

**Figure 2:**
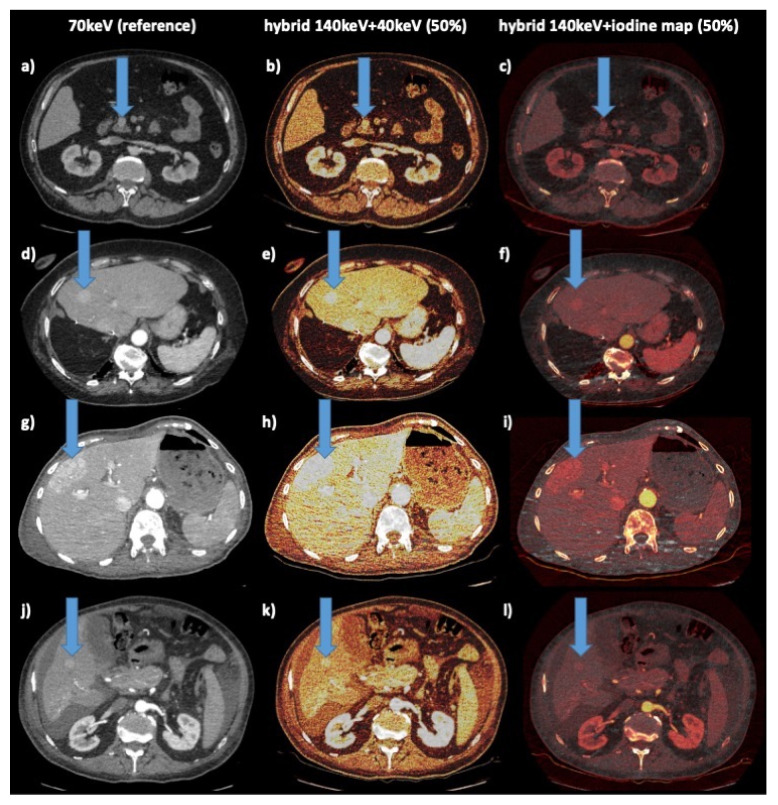
Example cases. Columns: left: reference 70 keV; middle: hybrid 140 kev–40 keV 50%; right: hybrid 140 keV iodine map 50% (color scheme: “hot iron”); arrow indicates liver lesion: 1st row (**a**–**c**): 61 y male with NEN of pancreatic head; 2nd row (**d**–**f**): 78 y female with NEN of ileum, hepatic metastasis, status post right liver resection; 3rd row (**g**–**i**): 75 y female with RCC, hepatic metastasis; 4th row (**j**–**l**): 61 y male with HCC, hepatic lesion, ascites, cirrhosis.

The study population consisted of 69 patients (women = 46% (32/69), men = 54% (37/69); mean age = 66 ± 12 years; age range = 27–87 years). A total of 70% of all hypervascular lesions were neuroendocrine neoplasms (48/69), 16% were hepatocellular carcinomas (11/69), and 14% were liver metastases from renal cell carcinoma (10/69) (Figure 1 and Figure 2). Maximum abdominal diameters were 255 ± 36 mm (sagittal) and 336 ± 37 mm (coronal). Tumor area was 1809 ± 2568 mm^2^.

### 3.2. Subjective Parameters

#### 3.2.1. Diagnostic Confidence

**Figure 3:**
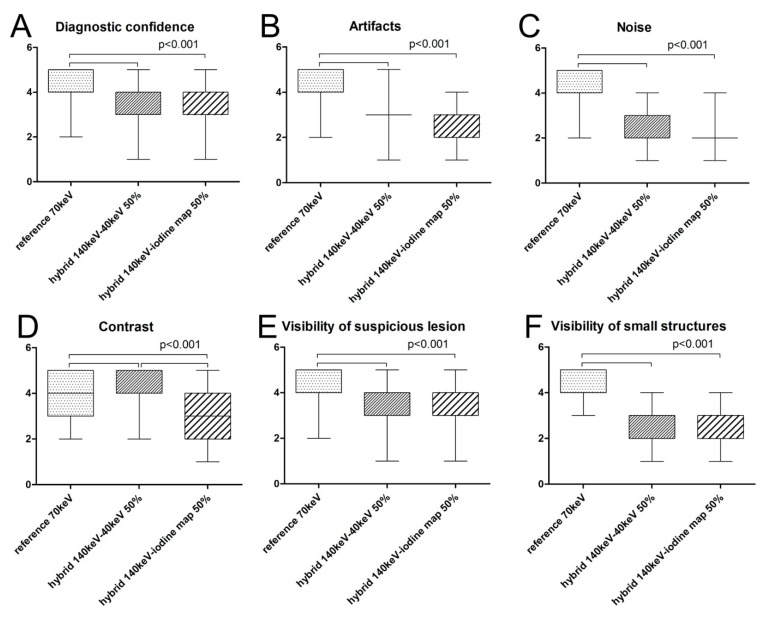
Boxplots of median scores for all subjective parameters for the three sets investigated. Median subjective Likert scores were highest for the 1st set, except for image contrast (**D**), for which the 2nd set was rated highest. For level of diagnostic confidence (**A**), artifacts (**B**), noise (**C**), and visibility of suspicious lesions (**E**) or small structures (**F**), scores for the 1st set were significantly higher than for the 2nd or 3rd set (*p* < 0.001). Regarding image contrast (**D**), the 2nd set was rated significantly higher than the 3rd set (*p* < 0.001), but the median did not differ significantly compared with the 1st set.

The median level of diagnostic confidence rated on a five-point Likert scale was highest for the 1st set (reference set with 70 keV). The median confidence score for this set was 5.0/1.0 versus 3.0/1.0 for the 2nd (hybrid 140 keV/40 keV set) and 3rd (hybrid 140 keV/iodine map set) set, which was significantly lower (*p* < 0.001). Between the 2nd and 3rd sets, median diagnostic confidence did not differ significantly (*p* > 0.05) (Figure 3).

#### 3.2.2. Artifacts

The median scores for presence of artifacts were highest for the 1st set (reference set with 70 keV). The median for the 1st set was 5.0/1.0 versus 3.0/1.0 for the 2nd (hybrid 140 keV/40 keV set) and 3rd (hybrid 140 keV/iodine map set), which was significantly lower (*p* < 0.001). Between the 2nd and 3rd sets, median scores did not differ significantly (*p* > 0.05) (Figure 3).

#### 3.2.3. Image Noise

The median noise scores were highest for the 1st set (reference set with 70 keV). The median for this set was 5.0/1.0. For the 2nd (hybrid 140 keV/40 keV set: 2.0/1.0) and 3rd (hybrid 140 keV/iodine map set: 2.0/1.0) sets, the median score was significantly lower (*p* < 0.001). Between the 2nd and 3rd sets, median scores did not differ significantly (*p* > 0.05) (Figure 3).

#### 3.2.4. Image Contrast

The median score for the level of contrast was highest for the 2nd set with 5.0/1.0 and was significantly higher than for the 1st set with 4.0/1.0 and the 3rd set with 3.0/2.0 (*p* < 0.001). The 1st and 3rd sets also differed significantly (*p* < 0.001) (Figure 3).

#### 3.2.5. Visibility of Suspicious Lesions

The median score for visibility of suspicious lesions was 4.0/1.0 for all three sets. However, because of different distributions, scores for the 1st set differed significantly from those for the 2nd and 3rd sets (*p* < 0.001), while scoring did not differ significantly between the 2nd and the 3rd set (Figure 3).

#### 3.2.6. Visibility of Small Structures

Median scores for the visibility of small structures were highest for the 1st set with 5.0/1.0 and were significantly higher than for the 2nd (2.0/1.0) and 3rd set (3.0/2.0) with *p* < 0.001. Median scores did not differ significantly between the 2nd and 3rd sets.

Kendall’s tau-b correlation showed high agreement between the two readers for all sets (Figure 3 and Table 2).

We found high agreement between the two readers for all sets.

### 3.3. Estimated Reading Time

Assuming the same reading intensity for all sets, we estimated a reading time for all possible datasets that was ten times higher compared to the sets investigated in this study (1st, a reference set with 70 keV; 2nd, a 50:50 hybrid 140 keV/40 keV set; 3rd, a 50:50 hybrid 140 keV/IM set) [15].

## 4. Discussion

DECT using VMIs has been reported to increase the detectability of hypervascular lesions [15,23,24,25,26]. Nevertheless, reading all possible postprocessed images is time-consuming, inefficient, and not feasible in the routine clinical setting [15]. Combining low- and high-energy levels may be beneficial regarding lesion detection as it provides high image contrast while ensuring acceptable noise levels. Therefore, hypervascular lesions seem to be the most suitable entities to investigate these issues. Our results show that, in principle, hybrid images offer the possibility to increase efficacy by lowering the reading time for assessing the additional information from SCT in clinical routine. At the same time, our results also revealed that the use of a rigid weighting did not improve diagnostic performance in hypervascular tumors in our study as the subjective rating results for the mono 70 keV reference set were superior to those for the other sets.

Several possible approaches have been proposed to efficiently extract additional information from DECT in clinical routine and thus overcome the problem that the reading of all image datasets is too time-consuming [15]. The most common method is to use the slope of HU in VMs. Another approach is to combine the advantages of different datasets by creating colored hybrid images [27]. Two combinations were evaluated in this study. While being more time efficient, the rigid hybrid images investigated by us did not improve diagnostic confidence in the assessment of hypervascular malignancies, as scoring was highest for the reference set using 70 keV. We tested our combinations in hypervascular lesions, for which the additional information obtained by DECT is potentially most useful, since absorption differences of the iodine contrast agent between the applied energy levels allow, among other things, quantification of iodine. Unless they are very small, hypervascular tumors are relatively easy to detect even on a standard CT scan. Furthermore, readers are more familiar with the appearance of these tumors in standard CT images. Taken together, this may explain the higher diagnostic confidence scores assigned to the reference set.

Nevertheless, contrast scores were highest for the 2nd set (140/40 keV, 50:50 blending) in our study. This is consistent with published results suggesting that low-energy levels generate higher image contrast and thus improve diagnostic confidence in the evaluation of hypervascular lesions [15,23,24,25]. However, since contrast of liver background tissue also increases, relative lesion contrast can also be reduced when fixed settings are used. Noise was rated highest in the 1st set, and the difference was significant compared with both hybrid sets. One possible reason for this result is the color overlay, which degrades subjective comparability of image noise.

In clinical routine, image preparation should ideally be performed by technical assistants, and on GE scanners, adjustment of superimposition ratios and windowing is only possible via the advantage workstation. Therefore, a comparable standard of reconstructed image sets is required for clinical routine, and appropriate standard parameters should be aimed for. The situation may be different for CT scanners from other manufacturers that allow real-time overlay within the PACS. In a study using CT datasets acquired on Siemens devices, Husarik et al. reported an added value for the imaging of both hyper- and hypoattenuating liver lesions using advanced virtual monoenergetic dual-energy levels ranging from 40 to 190 keV [28]. This was not considered in our study as no CT examinations performed on a Siemens CT scanner were available for comparison. In another study, Altenbernd et al. investigated image quality and detectability of hypovascular liver metastasis by DECT also performed on a Siemens dual-source multi-detector CT unit using different adjusted window settings. The authors conclude that dedicated window settings have a relevant effect on lesion conspicuity [29]. Furthermore, standardized imaging parameters may pave the way for artificial intelligence-based algorithms for image postprocessing, which may reduce reading time and thus increase cost-effectiveness.

There is no objective parameter comparability with overlays; therefore, they can only be used as a search series, similar to hybrid images in positron emission tomography (PET). Once a lesion has been identified, measurement of iodine concentration, which is independent of the hybrid image, can be performed and used in the same way as standard uptake values (SUVs) in PET. However, this would again involve additional effort as there are also differences in iodine concentration measurements between devices/vendors and limitations regarding low concentrations [30,31]. As inter-reader agreement was very good to excellent for all parameters in all sets investigated here, ratings seem to be valid [22].

Our study has some limitations. First and foremost, it was not possible to blind readers due to obvious differences between the image sets investigated. Second, lesions were quite large, and detectability was already good in the reference set. Third, readers were aware of the study design, which may have introduced a detection bias.

## 5. Conclusions

In clinical practice, SCT has the potential to improve lesion detectability and diagnostic confidence. Hybrid images may offer the possibility to efficiently assess the additional information provided by SCT. Nevertheless, in our study, the use of rigid blending did not improve diagnostic performance in hypervascular liver tumors. Calibrating a suitable detection image set, though, may potentially increase detectability while shortening reading time. Future work should therefore aim at identifying and defining optimal parameter settings to integrate the technique into the daily clinical workflow.

## Figures and Tables

**Table 1:** Technical CT scan parameters.

Phase	Arterial (GSI)	Portal Venous	Venous
Voltage	Dual-energy spectral mode (80/140 kVp)	Standard monoenergy mode (120 kVp)	
Postprocessing datasets	1st: 70 keV (reference) 2nd: hybrid 140 keV/40 keV (50:50 blending) 3rd: hybrid 140 keV/iodine map (50:50 blending)	Standard polychromatic images	
Recon. ASIR-V level	70%		
Noise index (NI)	21		
Pitch	1.375		
Collimation	64 × 0.625 mm		
Rotation time	0.7 s		
Tube current	average: 260–640 mA	min/max: 100/500 mA	
Smart mA	on		
Auto mA	off (not available from vendor)	on	
Recon mode	slice (axial)		
Recon slice thickness	0.625 mm		
Recon section interval	0.625 mm		
Field of view	DFOV: depending on patient SFOV: 50 cm		

**Table 2:** Inter-reader correlation calculated using Kendall’s tau-b test for all subjective parameters.

	Confidence	Artifacts	Noise	Contrast	Suspicious Lesions	Small Structures
**1st Set** Kendall’s tau-b *p*-value	0.833 <0.001	0.760 <0.001	0.848 <0.001	0.812 <0.001	0.806 <0.001	0.940 <0.001
**2st Set** Kendall’s tau-b *p*-value	0.862 <0.001	0.841 <0.001	0.701 <0.001	0.972 <0.001	0.887 <0.001	0.693 <0.001
**3st Set** Kendall’s tau-b *p*-value	0.909 <0.001	0.860 <0.001	0.814 <0.001	0.914 <0.001	0.818 <0.001	0.828 <0.001

## Data Availability

Data are available upon request from the corresponding author.
